# The development and validation of the daily electronic Endometriosis Pain and Bleeding Diary

**DOI:** 10.1186/1477-7525-8-64

**Published:** 2010-07-02

**Authors:** Linda S Deal, Dana Britt DiBenedetti, Valerie SL Williams, Sheri E Fehnel

**Affiliations:** 1 Patient Reported Outcomes, Pfizer, 500 Arcola Road, Collegeville, PA 19426, USA; 2 Patient Reported Outcomes, RTI Health Solutions, 3040 Cornwallis Road, PO Box 12194, Research Triangle Park, NC 27709-2194, USA

## Abstract

**Background:**

The objective of this study was to develop and validate a daily electronic Endometriosis Pain and Bleeding Diary (EPBD) for assessing treatment-related changes in endometriosis symptoms from the patient's perspective in a clinical trial setting.

**Methods:**

The EPBD items were developed based on clinician input and the results of 5 focus groups (N = 38) and 3 iterative sets of cognitive interviews (N = 22). The psychometric properties were evaluated using data collected in a usual-practice, non-intervention study conducted at 4 sites in the United States. Existing questionnaires were also administered to explore the construct validity of the EPBD. The development and validation processes were consistent with the recommendations in the 2009 FDA Patient Reported Outcomes Guidance to Industry.

**Results:**

Focus group participants described 2 distinct types of pain (intermittent and continuous), which they felt were relevant and important to monitor. Participants also indicated that pain and bleeding/spotting associated with intercourse were important symptoms related to endometriosis. Cognitive interviews with additional endometriosis patients served to optimize item content, wording, and response options. Psychometric analyses found the EPBD items to behave as expected, for example, item-level means for subjects with severe endometriosis symptoms were higher (i.e., worse) compared with subjects with mild symptoms. Item-total correlations for the EPBD pain items (range 0.40-0.89) indicated that the items were related but not redundant. EPBD pain ratings correlated highly with the modified Brief Pain Inventory-Short Form Pain Intensity score (range 0.46-0.61). Women with severe endometriosis symptoms reported significantly higher intermittent and continuous dysmenorrhea and intermittent and continuous pelvic pain ratings and greater interference with daily activities compared with women with mild symptoms (all p < 0.01).

**Conclusions:**

The results of this study show that the 17-item EPBD reliably and validly characterizes the types of pain that endometriosis patients identified as being important. As a daily patient-reported assessment, it overcomes the significant potential for intra- and inter-rater variability and rater and recall bias that is inherent in the Biberoglu and Behrman Scale. Additional studies are required to confirm the dimensionality and optimal scoring of the EPBD, to corroborate the present results, and to assess other important measurement properties, such as responsiveness.

## Background

Endometriosis is a common, chronic disorder that affects more than 5.5 million women in North America[1] and more than 70 million worldwide [2]. An estimated 2-10% of women of reproductive age have endometriosis [1]. Several studies have shown that endometriosis is associated with a significant economic and social burden [2-5], with hospitalizations, especially those related to surgical intervention, being the main direct cost-drivers [2,4]. Indirect costs include impaired health-related quality of life, diminished psychological and social functioning [2,6,7], and lost work productivity and earned income, all primarily due to pain [2].

The clinical symptoms of endometriosis include severe dysmenorrhea (painful menstruation), deep dyspareunia (pain with intercourse), chronic pelvic pain, ovulation-related pain, heavy menstrual bleeding and/or spotting between periods, and painful bowel and/or bladder symptoms that occur during or prior to menstruation [1]. The pain associated with endometriosis has little relationship to the type or location of the laparoscopically visible lesions [8]. It has been estimated that 30-40% of women with endometriosis have some degree of infertility [1,9]. The diagnosis of endometriosis is a histologic one that can only be achieved through invasive procedures (laparoscopy and excisional biopsy) [10]. Further complicating this disorder is the fact that there is often a significant delay between the onset of the symptoms of endometriosis and diagnosis [11,12]. This delay occurs at multiple levels and is associated with significant psychological and physical burden [12].

No fully validated instrument is currently available to assess endometriosis symptoms from the patient's perspective. The Biberoglu and Behrman (B&B) [13] Scale, the most commonly used standard for assessing endometriosis symptoms in a clinical setting, is limited by potential recall bias resulting from its use of a 4-week reference period. In addition, as a clinician-administered instrument, it is subject to rater bias, as well as both inter- and intra-rater variability. Although Ling and colleagues [14] addressed issues with the B&B Scale by having patients report directly on pelvic pain, dysmenorrhea, and dyspareunia daily using a 0 to 10 numeric rating scale (NRS), no qualitative research involving patient input to support the item concept and response scale selection was conducted. Finally, while the Endometriosis Health Profile-30 (EHP-30) [15-17] has been validated for use in assessing patient-reported well-being and functioning associated with endometriosis, it does not directly assess endometriosis symptoms. In addition, like the B&B Scale, it relies on a 4-week recall.

Patient-reported outcome (PRO) instruments are increasingly being used in clinical practice and clinical trials as a means to measure the benefits of treatment for which the patient is the sole or primary source of information on symptom change. In December 2009, the United States (US) Food and Drug Administration (FDA) issued a guidance on the development and use of PROs [18] to ensure that they are reliable and interpretable, that they measure what they are intended to measure, and that they are backed by a solid, scientific rationale.

The objective of this study was to develop and validate a daily electronic Endometriosis Pain and Bleeding Diary (EPBD) for assessing treatment-related changes in endometriosis symptoms from the patient's perspective. The diary was designed to be used in a clinical trial setting. The development and validation processes were consistent with the recommendations in the FDA Patient Reported Outcomes Guidance to Industry.

## Methods

This study was reviewed and approved by the Internal Review Board at the participating centers. Appropriate ethics committee approvals were obtained prior to any subject's participation in either the qualitative or quantitative phase of the study. All study participants provided written informed consent.

### Questionnaire Development (Qualitative)

The EPBD was developed using a qualitative process that included clinician input, focus groups, and cognitive interviews. Symptom concepts and response scale options for the EPBD were derived from a series of 5 focus groups comprised of women with endometriosis. Results from the focus groups and a search of the relevant literature were combined with input from a panel of clinicians specializing in the treatment of endometriosis and chronic pain to develop a draft set of diary questions and response scale alternatives addressing endometriosis symptoms that were meaningful and relevant to patients. The draft items were then subjected to 3 iterative rounds of cognitive interviews to test their comprehensiveness and relevance, to determine whether any items required revision or elimination, and to identify optimal response scales. The EPBD was refined following each round of interviews.

The inclusion criteria for the focus groups and cognitive interviews were similar. Participants were required to have been laparoscopically diagnosed with endometriosis within the past 5 years, be aged 18 to 45 years, and to have self-reported moderate to severe pain, (determined at screening by the B&B Symptom Scale), which they associated with their endometriosis and did not occur exclusively during menstruation. Women treated surgically for their endometriosis within the previous 6 months and those who reported complete pain relief from over-the-counter or prescription NSAIDs were excluded.

**Figure 1:**
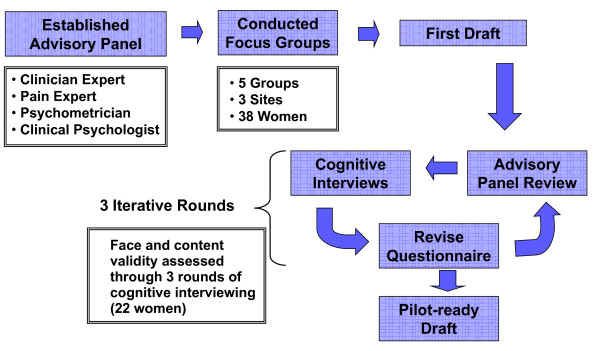
**Qualitative EPBD Development Process**. The EPBD development process.

Figure 1 illustrates the EPBD qualitative development process.

### Psychometric Evaluation (Quantitative)

#### Study Design

Psychometric evaluation was accomplished by administering the EPBD during a usual-practice, non-intervention study conducted at 4 sites in the United States. Participants continued their currently prescribed treatments; no additional study medications or other interventions were administered. The objectives of the study were to assess the measurement properties of the EPBD, including structure and scoring, internal consistency reliability, test-retest reliability, and construct and discriminant validity; and to evaluate the ease of use of the electronic EPBD (administered on a data capture device based on the Palm Pilot platform called the LogPad^® ^System [PHT, Corp., Charlestown, Massachusetts, USA]).

#### Study Population

Non-pregnant, non-lactating women between the ages of 18 and 45 with laparoscopically diagnosed endometriosis and mild or severe endometriosis symptoms were eligible to participate in the study. To facilitate the evaluation of discriminating ability, an interview script based on symptoms from the B&B Scale, was developed and administered at screening to prospectively assign participants to distinct known symptom severity groups (mild or severe). Subjects were required to have had regular menstrual cycles (21-35 days) for the past 3 months and to be able to read and understand English. In addition, they had to have engaged in sexual intercourse or other sexual activity involving full vaginal penetration within 30 days of screening, or have avoided sexual activity due to pain, or not have been sexually active because they lacked a partner, but would otherwise have been sexually active. No more than 20% of study participants were not sexually active due to lack of a partner. The use of leuprolide acetate or continuous-use oral contraceptives was permitted only for subjects who were still having monthly periods. Subjects who had undergone a hysterectomy or bilateral oophorectomy, those who had received surgical treatment for endometriosis within 1 month of screening, and those who were unable to use the electronic device were not eligible.

#### Clinical Assessments and PRO Measures

Clinical and demographic data were collected at baseline. Clinical data included date and method of endometriosis diagnosis; date of any surgical treatments for endometriosis; date of last menstrual period and information on the regularity of menstrual periods; pregnancy and lactation history; information on current sexual activity; current endometriosis treatments; and a brief medical history.

The following assessments were administered or self-completed during the study:

The symptom items of the B&B Scale were assessed during screening, at study visit 1 (baseline), and at the end of the study. The B&B Scale assesses the severity of the signs (pelvic tenderness, induration) and symptoms (dysmenorrhea, deep dyspareunia, and pelvic pain) of endometriosis over a 4-week period using a 4-point rating scale. An interview script was used by study coordinators to minimize rater variability for categorizing subjects as experiencing mild or severe symptoms. Higher scores indicate greater levels of pain (or worse symptoms).

Patients completed an electronic version of the EHP-30 at baseline and at the end of the study. The core EHP-30 comprises 30 items and uses a 4-week time reference to assess 5 multiple-item subscales (control and powerlessness, emotional well-being, pain, self-image, and social support). Higher scores indicate poorer health status.

A modified version of the Brief Pain Inventory - Short Form (mBPI-SF) [19], was administered at baseline and at the end of the study. The BPI-SF, originally developed to assess cancer pain, measures pain intensity, the impact of pain on daily functions, pain location, and analgesic use. With the author's permission, the pain location and analgesic use items were excluded from the BPI-SF used in this study (mBPI-SF). The mBPI-SF uses a 0 to 10 NRS to rate pain intensity (4 items), pain relief (1 item), and level of pain interference (7 items) from the patient's perspective. In the current study, the 4 severity items were averaged to assess pain intensity. The 7 items relating to pain interference were averaged to provide an overall interference score.

The electronic EPBD was self-completed each evening for approximately one menstrual cycle. A 24-hour reference period was selected to minimize recall bias and because focus group participants indicated that symptoms vary on a daily basis. Nine of the EPBD items require the respondent to choose either "yes" or "no" with the selected response routing subjects to subsequent questions according to a predetermined logic. Five items use a 0 to 10 NRS to describe either pain severity (0 = no pain to 10 = worst pain imaginable) or the level of interference caused by endometriosis pain (0 = did not interfere at all to 10 = interfered completely). Three items require the respondent to enter information concerning the frequency or duration of pain episodes.

#### Analytic Techniques

The distribution of responses and the extent of missing data for the 5 EPBD items that use a NRS were examined to identify potential response anomalies, such as floor or ceiling effects. Descriptive statistics were calculated for the overall sample and the mild and severe symptom subgroups.

Exploratory factor analysis, principal component analysis, and correlational analyses were used to characterize the structure of the EPBD and to determine the scoring algorithm. The internal consistency of the EPBD items was evaluated using Cronbach's[20] coefficient alpha and item-level data from each patient's initial and final assessment. The test-retest reliability of individual questionnaire items was estimated using intraclass correlation coefficients (ICCs).

Correlational analyses were conducted to examine the construct validity of the EPBD and its individual symptom items. Pearson correlations between average daily EPBD scores over a menstrual cycle and other measures were computed using data from the final clinic visit. EPBD ratings were expected to correlate relatively highly with the other analogous measures of symptom severity, such as the B&B Symptom Scale ratings, EHP-30 pain subscale, and mBPI-SF pain intensity. More specifically, it was expected that EPBD pain severity ratings would correlate more highly with the EHP-30 pain score than with the EHP-30 social support, control and powerlessness, emotional well-being, and self-image scores, and also more highly with the mBPI-SF pain *intensity *score than with the mBPI-SF *interference *score. Similarly, a higher correlation was expected between the EPBD pain interference rating and the mBPI-SF interference score compared to the lower correlations expected between the EPBD pain severity ratings and the mBPI-SF interference score. Known-groups analyses were conducted to determine the discriminating ability of potential EPBD scores. Hypothesis tests (t-tests) examined mean EPBD differences across comparison groups of interest, in particular, it was hypothesized that women with severe endometriosis symptoms would have worse (i.e., higher) EPBD pain severity ratings and interference scores compared to women with mild symptoms.

Only data for patients who completed the diary for at least 80% (or 25 days) of the menstrual cycle were included in the analyses. Scores for existing instruments were computed using guidelines published by the developers. The sample size determination for the quantitative phase of the study was based on the methods described by MacCallum [21]. All statistical tests are two-tailed. A type 1 error rate of 5% (alpha = 0.05) was applied to each hypothesis test. An error rate of 1% (alpha = 0.01) was applied to tests of correlation coefficients. All analyses were conducted using SAS Version 9.1 (SAS Institute, Inc. Cary NC 2005).

## Results

### Qualitative

A total of 38 women ages 20 to 45 years participated in the focus groups. Of these, 84% had been formally diagnosed with endometriosis within the last 2 years. The majority of participants (n = 33) reported being sexually active; of these, 18 women reported moderate pain, 14 reported severe pain, and one described her pain as moderate/severe. Of the 5 women not reporting current sexual activity, 3 reported avoiding intercourse due to endometriosis.

The focus group participants described 2 distinct types of pain (intermittent and continuous), which they felt were relevant and important to measure. Intermittent pain was described by participants as sudden and "sharp shooting" pain, while continuous pain was described as "dull ache" or "aching" and longer lasting. Participants also indicated that pain and bleeding/spotting associated with intercourse were important symptoms related to endometriosis. All participants agreed that a 0 to 10 NRS would be appropriate to rate changes in pain over time. After completing 5 focus groups, no new symptom or severity-level measurement ideas were introduced (concept saturation was achieved), indicating that the items contained in the EPBD were relevant to women with endometriosis and consistent with how they view their symptoms.

The draft EPBD items were subjected to 3 iterative rounds of cognitive testing with 22 additional endometriosis patients to optimize diary content, item wording, and response scales. Participants in the cognitive interviews also provided important information about their interpretation of the questions, as well as their approaches to the response process. After completing 3 rounds of interviews and revisions, the resulting EPBD was comprised of 17 items.

### Quantitative

A total of 128 women (ages 18 to 45; mean 33.9 years) participated in the non-intervention validation study. Of these, 60 (46.9%) had mild endometriosis symptoms and 68 (53.1%) had severe endometriosis symptoms (as determined at screening by the B&B Symptom Scale interview script). The compliance rate for completing the electronic EPBD was 90%.

#### Descriptive Statistics

In all cases, the item means for subjects with predetermined severe endometriosis symptoms were worse (i.e., higher) compared with subjects with predetermined mild symptoms. Although there was no evidence of distributional anomalies for any of the EPBD items, the responses were somewhat sparse toward the upper ends of the distributions. As would be expected, this was particularly true in the mild endometriosis symptom group. The largest percentage of missing values for any item not related to sexual intercourse was 12.5% (n = 16 missing) at day 25 for worst continuous pain. The rates of missing data seen for items related to sexual intercourse ranged from 4.7% to 90.6%.

#### Structure and Scoring

The principal components and factor analysis results did not support separate scoring of intermittent and continuous endometriosis pain, but instead pointed to a single dimension underlying the severity of endometriosis pain. Five EPBD pain ratings (intermittent pelvic pain, continuous pelvic pain, intermittent dysmenorrhea, continuous dysmenorrhea, and dyspareunia) were scored and analyzed separately to accommodate comparison to clinical terminology and the B&B Symptom Scale items. Daily ratings were averaged over the menstrual cycle to obtain each woman's EPBD scores. For all NRS questions, days without pain were scored as zero.

Item-total correlations ranged between 0.40 and 0.89, indicating that the EPBD items are each related to the other items, without being redundant (Table 1).

**Table 1:** Item-total Correlations

EPBD Item	Day 1	Day 7	Day 14	Day 21	Day 25
Worst intermittent pain	0.79	0.65	0.72	0.66	0.70
Episodes of intermittent pain	0.56	0.59	0.58	0.52	0.54
Duration of intermittent pain	0.52	0.46	0.40	0.51	0.55
Average continuous pain	0.89	0.78	0.83	0.84	0.81
Worst continuous pain	0.87	0.80	0.83	0.81	0.81
Duration of continuous pain	0.62	0.64	0.68	0.60	0.66
Pain interference	0.81	0.78	0.82	0.77	0.83

EPBD = Endometriosis Pain and Bleeding Diary

#### Reliability

##### Internal Consistency

The internal consistency reliability of the EPBD items was acceptable to good. Cronbach's alpha was 0.83 for the initial assessment and 0.73 for the final assessment for continuous pain compared with 0.62 and 0.58 for intermittent pain. The internal consistencies for items assessing continuous pain were higher than those for intermittent pain, and the internal consistencies for items assessing dysmenorrhea were higher than those for pelvic pain (that is, endometriosis pain in the absence of bleeding) (Table 2).

**Table 2:** Internal Consistency Reliabilities

	Initial Assessment	Final Assessment
Dysmenorrhea - Intermittent and Continuous	0.81	0.84
Pelvic Pain - Intermittent and Continuous	0.55	0.63
		
Intermittent pain	0.62	0.58
Dysmenorrhea - Intermittent	0.64	0.72
Pelvic Pain - Intermittent	0.54	0.52
		
Continuous Pain	0.83	0.73
Dysmenorrhea - Continuous	0.86	0.79
Pelvic Pain - Continuous	0.77	0.68

##### Test-Retest

The ICCs for test-retest reliability for women with dysmenorrhea were acceptable for the NRS pain items of the EPBD (range 0.65-0.72). The test-retest reliability results for the NRS pain items for women with pelvic pain symptoms were also acceptable (range 0.59-0.69) (Table 3). Test-retest reliabilities for dyspareunia were not interpretable due to the small sample size.

**Table 3:** Test-Retest Intraclass Correlation Coefficients: EPBD Numeric Rating Scale Items

EPBD NRS Items	Dysmenorrhea	Pelvic Pain
Worst intermittent pain	0.69	0.69
Average continuous pain	0.72	0.59
Worst continuous pain	0.65	0.62
Pain interference	0.56	0.71

EPBD = Endometriosis Pain and Bleeding Diary; NRS = numeric rating scale

#### Validity

The correlations between the EPBD and the B&B Symptom Scale ratings were generally lower (range 0.15-0.54) than the correlations between the EPBD and EHP-30 (range 0.26-0.65) and mBPI-SF (range 0.34-0.73). Not all correlations between the EPBD and B&B Symptom Scale were statistically significant, while all correlations between the EPBD and other measures were statistically significant and sizeable (Table 4).

**Table 4:** EPBD Validity Correlations

	Pelvic Pain		Dysmenorrhea		Dyspareunia	Pain Interference
	Intermittent	Continuous	Intermittent	Continuous		
**Visit 2 B&B**						
Pelvic Pain	0.20	0.35^‡^	0.19	0.32^†^	0.27*	0.39^‡^
Dysmenorrhea	0.20	0.28*	0.29*	0.29*	0.21	0.32^†^
Deep Dyspareunia	0.28*	0.36^†^	0.24	0.15	0.54^‡^	0.31*

**Visit 2 EHP-30**						
Control/Powerlessness	0.38^‡^	0.45^‡^	0.33^†^	0.38^‡^	0.30*	0.52^‡^
Emotional Well-being	0.29*	0.35^‡^	0.28*	0.28*	0.27*	0.48^‡^
Pain	0.42^‡^	0.54^‡^	0.44^‡^	0.56^‡^	0.41^‡^	0.65^‡^
Self-Image	0.27*	0.34^†^	0.26*	0.32^†^	0.26*	0.44^‡^
Social Support	0.37^‡^	0.38^‡^	0.35^‡^	0.28*	0.32*	0.46^‡^

**Visit 2 Modified BPI-SF**						
Interference	0.51^‡^	0.59^‡^	0.50^‡^	0.47^‡^	0.34^†^	0.73^‡^
Intensity	0.61^‡^	0.61^‡^	0.56^‡^	0.55^‡^	0.46^‡^	0.70^‡^

EPBD = Endometriosis Pain and Bleeding Diary; B&B = Biberoglu and Behrman Scale;

EHP-30 = Endometriosis Health Profile-30; BPI-SF = Brief Pain Inventory - Short Form

*p < 0.01, ^†^p < 0.001, ^‡^p < 0.0001

The correlations between the EPBD ratings and EHP-30 subscale scores were mostly moderate to large. The EPBD pain ratings were more highly correlated with the EHP-30 pain score (range 0.41-0.65) than with the other domains measured by the EHP-30 (range 0.26-0.52), as hypothesized. The EPBD pain interference rating correlated most highly with all EHP-30 subscores (range 0.44-0.65) (Table 4).

EPBD pain severity ratings and mBPI-SF intensity scores were highly correlated (range 0.46-0.61). Slightly lower, but still significant (p < 0.01), correlations were noted between the EPBD pain ratings and the mBPI-SF interference score (range 0.34-0.59). The correlation between the EPBD pain interference item and the mBPI-SF interference score (0.73) was slightly greater than the correlation between the EPBD pain interference item and the mBPI-SF intensity score (0.70) as expected (Table 4).

Women with severe endometriosis symptoms reported significantly (p < 0.001) greater intermittent and continuous dysmenorrhea and intermittent and continuous pelvic pain ratings than women with mild symptoms (Table 5). Women with severe symptoms also reported significantly greater interference with daily activities.

**Table 5:** Known Groups Analyses Examining EPBD Discriminating Ability: Mild versus Severe Symptom Groups

Average Daily EPBD Pain Rating	Mild Symptoms Mean (SD)	Severe Symptoms Mean (SD)	*t*
Pelvic Pain - Intermittent	1.21 (1.4), n = 60	2.00 (1.8), n = 68	-2.70*
Pelvic Pain - Continuous	0.89 (1.3), n = 60	2.06 (2.1), n = 68	-3.80^†^
Dysmenorrhea - Intermittent	1.76 (1.6), n = 58	3.19 (2.2), n = 66	-4.13^‡^
Dysmenorrhea - Continuous	2.40 (2.0), n = 58	3.90 (2.5), n = 66	-3.70^†^
Dyspareunia	1.69 (1.9), n = 49	2.68 (2.4), n = 53	-2.29
Pain Interference	0.85 (1.0), n = 60	1.91 (1.8), n = 68	-4.16^†^

EPBD = Endometriosis Pain and Bleeding Diary; SD = standard deviation.

*p < 0.01, ^†^p < 0.001, ^‡^p < 0.0001

## Discussion

The present study provides important results regarding the content validity and measurement properties of the EPBD. The EPBD overcomes the shortcomings of existing instruments in that it is assessed daily and directly by the patient. It is an improvement on Ling and colleague's 0 to 10 NRS in that it allows for the qualitative distinction between intercourse avoidance and the most painful intercourse possible. Using the Ling scale, both scenarios are rated a value of 10. In addition, our qualitative research involving patient input supports the item content. The use of qualitative research involving patient input is heavily emphasized in the FDA PRO Guidance to Industry.

Descriptive results showed no evidence of distributional anomalies or response biases. The highest rates of missing data were observed for items related to sexual intercourse. We expected that these items would have the highest rate of missing data for two reasons: this was a non-intervention study in which women who were avoiding sexual intercourse due to pain likely continued to do so; and the study included women without sexual partners, who would not be able to report on current sexual activity.

Although the correlational and factor analyses indicated that endometriosis-associated pain severity is unidimensional and internally consistent, the ratings for intermittent and continuous pain were not combined for scoring because focus group and cognitive interview participants strongly indicated that this distinction is important to patients. Furthermore, maintaining the separation of these items is consistent with FDA guidance to industry on content validity. In the focus groups, women indicated that the majority of their endometriosis-associated pain occurred during the few days prior to and several days into their menstrual periods, but spoke about this pain collectively as pain related to their periods, rather than distinguishing between pain with and without bleeding. While pain type distinctions related to the absence or presence of bleeding have clinical relevance, data from this study suggest that the distinction between dysmenorrhea and pelvic pain associated with endometriosis may not be important from the patient's perspective.

While item-level test-retest reliability was variable, the reliabilities of the 0 to 10 NRS endometriosis pain symptom and interference ratings were generally satisfactory. Subsets of EPBD items demonstrated acceptable internal consistency reliabilities. Item-total correlations indicated that the EPBD items were appropriately interrelated without being redundant.

The correlations between the EPBD and other measures of pain and endometriosis provide support for the construct validity of the EPBD. As expected, the EPBD pain ratings were most highly correlated with other patient-reported measures of pain and the impact of endometriosis symptoms (i.e., the mBPI-SF pain intensity score and the EHP-30 pain subscale) and less correlated with the clinician-administered B&B Symptom Scale. The lower correlations between the EPBD and the B&B Symptom Scale ratings for all items except the EPBD dyspareunia rating and the B&B Symptom Scale deep dyspareunia score are likely due to the limitations of the B&B Symptom Scale which employs a 4-week recall period and is interviewer-assessed, while the EPBD is an unfiltered self-report. Also expected was the higher correlation between the pain interference scores on the EPBD and the mBPI-SF compared with the correlation between the EPBD pain *interference *and the mBPI-SF *intensity *score. This provides support for the divergent validity of the EPBD ratings, i.e., regardless of whether the concepts are measured using the mBPI-SF or the EPBD pain interference is related to but not the same as pain intensity/severity.

The EPBD successfully differentiated patients with severe and mild endometriosis symptoms, thereby providing preliminary support for the discriminating ability of the EPBD. Women with severe symptoms also reported significantly greater interference with daily activities. While not a direct measure of responsiveness, these results suggest that the EPBD pain severity ratings will be sensitive to treatment-related improvements in clinical trials.

The results of this study indicate that the EPBD is a useful measure of symptoms that are relevant for patients with endometriosis, that is, it reliably and validly characterizes the different types of endometriosis pain identified by patients in early qualitative research that laid the groundwork for the development and content of the EPBD. These are intermittent pelvic pain, intermittent dysmenorrhea, continuous pelvic pain, continuous dysmenorrhea, and dyspareunia. Because it is a patient-reported daily assessment, the EPBD overcomes the significant potential for intra- and inter-rater variability and rater and recall bias that is inherent in the B&B Scale. The 90% compliance rate for EPBD completion on the electronic device suggests that the technology was sufficiently simple for subjects to use.

The limitations of this research are concentrated in the quantitative phase and a result of the study design and study population. Because the validation study was non-interventional, we were unable to evaluate the sensitivity of the EPBD to detect treatment-related changes in symptoms, i.e., responsiveness. Additionally, we were unable to conduct known-groups validity analyses to provide support for the EPBD's ability to discriminate between women undergoing efficacious treatment for endometriosis symptoms compared with women receiving a placebo. We were also limited in our ability to fully evaluate dyspareunia due to a small sample size of sexually active women and women with sexual partners throughout the study. Our study sample included some women who avoided sexual intercourse due to pain, and because the study design was non-interventional, these women likely continued to avoid sexual intercourse throughout the study. Finally, we believe that the study would have benefited from a larger overall sample size with a more diverse geographic and ethnic representation.

The next step in documenting the validity evidence for the EPBD is to confirm the present results, verify the dimensionality of the EPBD and its optimal scoring algorithm, more thoroughly evaluate the validity of the dyspareunia symptom rating, and assess other important measurement properties, such as responsiveness. This will require a double-blind comparator-controlled (active or placebo) intervention study design. In addition validation of the dyspareunia scores will require including women who have a consistent opportunity to report on pain experienced with intercourse. Efforts to recruit a diverse geographic and ethnic sample to confirm the appropriateness of the symptoms experienced as reflected in the EPBD across cultures are also important.

Pfizer will make non-exclusive licensing agreements available to individual researchers and private practitioners who wish to use the EPBD. These licenses will include the instructions, questions, response scales, branching logic, and a conceptual framework. The EPBD has been developed and psychometrically evaluated for use in an electronic format. The transference and implementation of the instrument content to an electronic format is the full responsibility of the licensee.

## Conclusions

To the best of our knowledge, the EPBD is the only daily patient-reported instrument developed from the perspective of the patient that assesses the most important symptoms that women associate with their endometriosis. The EPBD may be useful to clinicians in assessing the impact of treatment on the symptoms reported by their patients with endometriosis. In particular, its discriminating ability may be useful in facilitating treatment decisions, as choice of treatment may be dependent upon symptom severity. Additionally, the EPBD is the only patient-reported instrument to assess intermittent and continuous pain, two very distinct but equally important types of pain that women with endometriosis report they experience.

## Competing interests

**Linda Deal, MS: **At the time this research was conducted Linda Deal was an employee of Wyeth, the sponsor of this study. Wyeth was acquired by Pfizer in October 2009. Ms Deal is now an employee of Pfizer and as part of her employment she now holds shares in Pfizer. The processing fees for this publication will be paid by Pfizer. No other financial or non-financial interests to declare.

**Dana Britt DiBenedetti, PhD: **No financial or non-financial interests to declare.

**Valerie S. L. Williams, PhD: **No financial or non-financial interests to declare.

**Sheri E. Fehnel, PhD: **No financial or non-financial interests to declare.

## Authors' contributions

Each author contributed substantially to the design of the study, the data analysis, and the development of the manuscript. Each has approved this submission.
